# Use of ultraviolet light in graduate medical education to assess confidence among residents and fellows in handwashing instruction

**DOI:** 10.1017/ash.2021.208

**Published:** 2022-04-20

**Authors:** Shaza Aouthmany, Haley Mehalik, Morgan Bailey, Mitchell Pei, Sameer Syed, Kristopher Brickman, Kayla Morrison, Sadik Khuder

**Affiliations:** 1 University of Toledo College of Medicine and Life Sciences, Toledo, Ohio; 2 Department of Biostatistics and Epidemiology, University of Toledo, Toledo, Ohio

**Keywords:** Hand Hygiene, COVID 19, Curriculum, Graduate Medical Education

## Abstract

**Background:**

Coronavirus disease 2019 (COVID-19) has made it imperative to focus on strategies to improve hand hygiene to minimize threats of viral transmission in hospitals.

**Objective:**

We investigated the potential of using ultraviolet (UV) light as a visual tool in hand-hygiene education to bring awareness of individual handwashing effectiveness to healthcare workers.

**Methods:**

In 2020, 117 individuals participated in the simulation and completed surveys on proper handwashing technique. Of these, 114 were first-year residents and fellows. Surveys of confidence in hand hygiene were obtained before and after formal hand-hygiene education utilizing UV light with Glo Germ lotion. The UV light and Glo Germ lotion were used to identify deficiencies in individual handwashing technique.

**Results:**

With a total response rate of 97.4%, first-year residents and fellows demonstrated a significant decrease in handwashing confidence in pre- and posteducation surveys. Study participants who had had formal hand-hygiene training in the previous 3 years also indicated confidence in hand hygiene similar to those who had not had previous hand-hygiene training. Conclusions: Overall, resident interns and fellows may have falsely elevated their hand-hygiene confidence levels. However, conclusions regarding the confidence of residents and fellows individually could not be made due to sample size. Many healthcare personnel practice improper handwashing techniques, which may be improved with education and training that includes UV light.

Even though maintaining proper hand hygiene has always been imperative in reducing transmission of pathogens, especially in the healthcare industry, its relevance has been reestablished due to the coronavirus disease 2019 (COVID-19) pandemic. Worldwide, thousands of deaths result daily from iatrogenic infections that are passed from healthcare workers to patients.^1^ Thus, maintaining proper hand hygiene is one of the most important and effective ways to reduce the transmission of pathogens and preventing deaths that result from this transmission.

Although our knowledge of severe acute respiratory coronavirus virus 2 (SARS-CoV-2) is evolving, the current literature reveals that the virus can be transmitted from infected patients to inanimate surfaces, where viral particles can remain active for up to 6 days and can infect individuals who come into contact with them. Alcohol-based hand rubs can lead to inactivation of the virus and thus prevent this process. Ethanol and 2-propanol are effective at inactivating SARS-CoV-2 at concentrations ≥30%. However, this activation occurs after 30 seconds of application, which is recommended but not regularly practiced.^2,3^ Healthcare professionals have low rates of compliance to proper hand-hygiene protocol, and physicians have the lowest compliance.^4^ In addition, even in those who have developed good hand-hygiene habits, handwashing quality is poor, especially in nails, fingertips, and skin between the fingers.^5^

An ultraviolet (UV) light can be utilized after application of a Glo Germ lotion (Moab, UT) to identify improperly cleaned areas during hand washing. Proper handwashing technique plays a major role in preventing pathogen transmission from provider to patients within our healthcare facilities and reducing hospital-acquired infections hospitalwide.^6^ UV light can be used to monitor surgical hand-rub technique in medical students, providing immediate feedback.^7^ This tool could be an effective method of identifying inadequate handwashing techniques within graduate medical education for our students and training residents in the healthcare system. Multifaceted techniques that merge the use of continued feedback of performance along with education and reinforcement can play a key role in handwashing compliance rates.^8^ Therefore, we evaluated 2 main hypotheses in this project: (1) Interns’ confidence in handwashing technique will be lower after an educational session that includes application of a Glo Germ lotion followed by hand washing. (2) Fellows’ confidence in handwashing technique will not be lower after an educational session that includes application of a Glo Germ lotion followed by hand washing.

A secondary goal of this project is to bring awareness to one of the most serious problems facing healthcare workers today: the spread of infections in medical facilities. Through our research, we hope to bring more concrete knowledge regarding the importance of proper handwashing techniques in preventing the spread of pathogens.

## Methods

### Setting and participants

This study was conducted at the University of Toledo Medical Center simulation center during the week of orientation at the start of the academic year in 2020. In total, 117 participants took part in the simulation; 114 were new interns, transfers, and fellows. All of the study participants were educated on infection control and volunteered to be a part of this study. All of the residents and fellows surveyed were eligible for study inclusion. Those who did not complete the survey were not included.

### Interventions

At the beginning of the study, prior to presentation on proper handwashing techniques, the study participants utilized a visual analogue scale to indicate how confident they were in washing their hands. The lowest score of 1 indicated not confident at all, and the highest score of 5 indicated extremely confident. Study participants used only whole numbers on this scale. Study participants were then given a presentation on proper handwashing techniques as indicated by the Centers for Disease Control and Prevention (CDC). Participants were then given Glo Germ lotion to apply to their hands. They were then instructed to wash their hands with soap and water. Afterward, participants’ hands were inspected under UV light. Areas that glowed were shown to the participants as areas they need to focus on washing. After the demonstration, participants completed the second part of the survey. Using the same visual analogue scale, they were asked to rank their confidence in their handwashing abilities after they used the Glo Germ lotion.

Data were collected using the World Health Organization (WHO) questionnaire called “Hand Hygiene Knowledge Questionnaire for Healthcare Workers,” which included demographics and reporting whether study participants had received formal hand-hygiene training in the previous 3 years and a quiz on currently accepted hand-hygiene practices.

### Outcomes measured

Participants received paper packets with a WHO questionnaire, initial survey, and final survey and submitted the packets anonymously at the end of the study. The outcomes measured included pre- and postsimulation handwashing confidence and participants’ accuracy on the WHO questionnaire quiz on currently accepted hand-hygiene practices. All participant responses were recorded and the data were compiled.

### Analysis of the outcomes

A descriptive statistical analysis of the data was conducted. Categorical variables were presented as frequencies and percentages, and descriptive statistics (mean ± standard deviation) were used for continuous variables. ANCOVA was utilized to determine the significance of the difference between initial and final handwashing confidence in participants who had had prior formal hand-hygiene training in the previous 3 years and those who had not.

The University of Toledo BioMed Committee approved this study. Informed consent was obtained prior to simulation. No identifiers or protected health information was used or recorded.

## Results

In total, 117 residents and fellows attended the session and 114 study participants completed the survey (response rate, 97.4%) before and after the hand-hygiene presentation. The demographics of the study population are displayed in Table 1. No significant patterns were detected regarding previous hand-hygiene training in study participants regarding different age groups, genders, professions, and departments.

**Table 1.** Demographics of 114 First-Year Residents and First-Year Fellows

	Prior Hand Hygiene Training		P-value
	No (n %)	Yes (n %)	
Age group			
25-29	21 (31.3)	46 (68.7)	0.172
30-50	19 (44.2)	24 (55.8)	
Gender			
Male	21 (32.3)	44 (67.7)	0.182
Female	21 (44.7)	26 (55.3)	
Profession			
Resident	40 (37.7)	66 (62.3)	0.989
Fellow	3 (37.5)	5 (62.5)	
Department			
Surgical	3 (15.8)	16 (84.2)	0.077
Non-surgical	25 (37.3)	42 (62.7)	

**Fig. 1.**
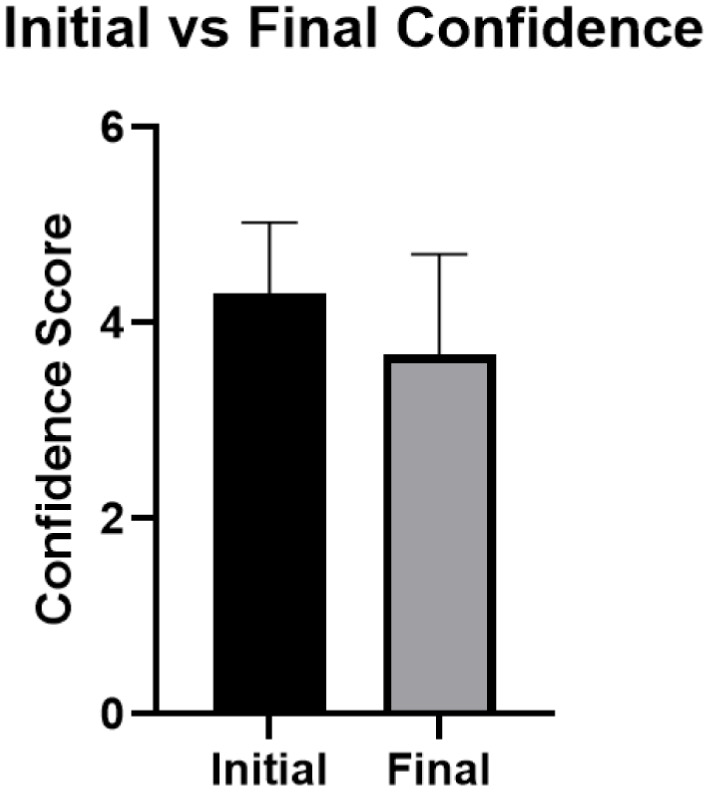
Mean hand washing confidence score before and after hand hygiene education.

The results for the confidence levels in Figure 1 indicate a significant decrease in mean confidence of handwashing capabilities of residents and fellows (N = 114; 95% CI; *P* < .0001; SD of differences, 1.236) after using UV light. The average initial handwashing confidence score (before the use of UV light) was 4.307 ± 0.718, and the average final handwashing confidence score (after the use of UV light) was 3.675 ± 1.026.

In Table 2, first-year residents and fellows who had previously had formal hand-hygiene training in the previous 3 years had similar differences between initial and final handwashing confidence levels compared to those who had not.

**Table 2.** Previous Hand Hygiene Training Effects on Study Participant’s Handwashing Confidence (Analysis of Covariance)

Source	*df*	Sum of Squares	Mean Square	F	*p*
Prior Hand Hygiene Training	1	1.75	1.75	1.15	0.2866
Error	112	170.78	1.52		
Corrected Total	113	172.53			

## Discussion

When comparing the confidence levels before and after handwashing simulation using UV light and Glo Germ, first-year residents and fellows had a significant decrease in confidence in their ability to eliminate the spread of pathogens using their current handwashing techniques. As shown in Figure 1, a statistically significant decrease in the mean handwashing confidence from 4.307 to 3.675, or 12%, was observed for the entire study cohort (N = 114; 95% CI; *P* < .0001). This finding suggests that medical residents and fellows falsely placed a high level of confidence in their ability to eliminate the spread of pathogens using their handwashing techniques before the study. Thus, a more intensive handwashing training session may be necessary. Particularly, targeted education efforts on technique are needed.^9^

Study participants were further stratified to those with and those without prior formal hand-hygiene training in the previous 3 years. Participants who had previous formal hand-hygiene training in the previous 3 years did not have a significant difference in confidence compared to those who did not have training. The indication that previous hand-hygiene training was not sufficient in increasing confidence of the study participants indicate the need for additional hand-hygiene training methods. Accordingly, the use of UV light can be used in hand-hygiene training as a physical reinforcement.

Furthermore, the correct answers to the WHO hand hygiene survey of each study participant could be used as a representative measure of their hand-hygiene knowledge and awareness; a higher score would indicate increased cognizance of proper hand-hygiene protocol and practice. Among our study participants, the mean percentage of correct responses was 59.83% overall, and it was 60.45% among those who indicated they have received prior hand-hygiene training. These low scores may indicate the necessity to improve the current methods being used to instruct proper hand hygiene among residents; perhaps the utilization of UV light can supplement hand-hygiene instruction in graduate medical education. For instance, one question from the WHO survey reads, “What is the minimum time needed for alcohol-based hand rub to kill most pathogens on your hand?” Although the correct answer was 20 seconds, only 62.28% of survey respondents answered this question correctly. Proper application and timing of sanitizer and soap are critical in removing potentially harmful substances from the skin surface. With UV light, the concept of identifying typically missed patterns along with immediate visual feedback has a crucial role to improving hand hygiene.^10^

This study had several limitations. We were unable to identify whether individuals who are further advanced in their medical career will show more accurate confidence levels due to their more advanced understanding of proper handwashing procedures. Handwashing confidence between residents and fellows individually could not be determined due to the difference in sample size. Further studies should be conducted to determine whether residents and fellows’ current handwashing methods are incorrect or are simply performed incorrectly. Studies could additionally be performed utilizing residents and fellows from other specialties.

In conclusion, UV light use after hand washing revealed that while residents and fellows in graduate medical education acknowledged the importance of hand washing in the hospital setting, many have a false sense of ability to properly wash hands. UV light may be used as an additional supplement to traditional hand-hygiene education to increase proper hand-hygiene compliance.
